# Summertime Characteristics of Atmospheric Polycyclic Aromatic Hydrocarbons in a Coastal City of Northern Poland

**DOI:** 10.3390/ijerph20054475

**Published:** 2023-03-02

**Authors:** Patrycja Siudek

**Affiliations:** Institute of Meteorology and Water Management—National Research Institute, Waszyngtona 42, 81-342 Gdynia, Poland; patrycja.siudek@imgw.pl

**Keywords:** PAHs, the gas phase, particulate matter, deposition fluxes, gas/particle partitioning

## Abstract

Parent polycyclic aromatic hydrocarbons (PAHs) in the gas and particle fraction were measured between May and August 2021 at a coastal urban site in Poland, to examine their chemical characteristics, distribution, sources, deposition fluxes and interactions with basic meteorological drivers. The mean concentration of PAHs in the gas phase was significantly higher (26.26 ± 15.83 ng m^−3^) than levels measured in the particle phase (1.77 ± 1.26 ng m^−3^). The highest concentration in the gas phase was found for phenanthrene (Phe), followed by fluoranthene (Flt), acenaphthene (Ace) and naphthalene (Naph). The contribution from each group of PAHs to the total particulate phase accounted for 50%, 25%, 14% and 12% for 3-, 4-, 5- and 6-ring compounds, respectively. The mean ΣPAH deposition flux was 59 ± 24 ng m^−2^ day^−1^. During the whole field campaign, the efficient removal of PM-bound PAHs was typically observed after precipitation events. Based on statistical analysis, it was found that 4-ring PAHs were less effectively removed (25%) by daily precipitation as compared to 5- and 6-ring components, whose fluxes decreased by 32% and 53%, respectively. This study revealed local urban sources such as vehicular emissions, coal-fired power plants, shipping activities, docks/ports infrastructure and municipal solid waste recycling units as predominant contributors to PM-bound and gas-phase PAHs.

## 1. Introduction

Polycyclic aromatic hydrocarbons (PAHs) are important organic compounds of the Earth’s climate system [1]. Atmospheric PAHs can exist in the gas phase and aerosol, which depends on their chemical structure and properties, but also complicated mechanisms of their formation/transformation [2,3]. Anthropogenic sources such as incomplete combustion of fossil fuel, industrial activities and traffic emissions can emit a substantial amount of volatile, semivolatile and non-volatile PAH compounds into the atmosphere over urban agglomerations [4,5]. In many urban areas, PAH emissions are of great concern due to their climatic, human health and environmental effects [6,7]. The spatial distribution of PAH levels in the atmosphere is mainly controlled by local emission sources, thus relatively lower concentrations can be observed away from major hotspots [8]. Extreme PM mass concentrations are often associated with high concentrations of PAH compounds in particulate fractions, which were identified in many areas in Europe [9,10,11] and Asia [12,13]. Moreover, higher concentrations of PAHs were found in winter than in summer [14]. For example, Yang et al. [15] showed significantly lower PAH concentrations during summer than winter, because of lower anthropogenic emissions and faster photochemical degradation rates. Many previous studies determined similar features, indicating significant anthropogenic, chemical and meteorological pathways for PAH seasonal profiles [9,10,11,12,13]. Morville et al. [9] showed that the most abundant components in the gas and particulate phase were naphthalene followed by phenanthrene and acenaphthene. They also found that diurnal variations of PAHs at the rural (Erstein), urban (Strasbourg) and suburban (Schiltigheim) sites in the NE France region during the summer period were determined by the intensity of vehicle traffic, with the highest concentrations around the morning rush hour and the evening [9]. Chimjarn et al. [16] observed 2.5-fold and 6-fold higher concentrations for the gas and particulate phases, respectively, during the cold season than in the warm season.

Summertime atmospheric processes of PAHs can include multiple pathways (i.e., soil-/water-air gas exchange, gas/particle partitioning, homogeneous reactions in the gas phase, heterogeneous reactions, oxidation by hydroxyl radicals (OH), NO_3_ radicals, and ozone (O_3_), transport, degradation, dry/wet deposition) [17,18]. For example, analysis of recent trends in urban Beijing showed an almost 20% higher contribution of 6-ring PAHs than 2–3 ring components during summer due to the effect of high ambient temperature on low molecular weight PAHs [19]. Gas/particle partitioning is a key mechanism responsible for the formation and transformation of secondary organic aerosols via a partition of PAH compounds between gas and particle fractions [15].

Few studies are available [10,20,21] that have investigated the distribution, fluxes, source apportionment, and meteorological effects on individual PAH components in different regions of Poland. However, only Siudek [22] has performed comprehensive research on parent PAHs in the gas and particle fraction over the Baltic Sea environment (N Poland) but without the inclusion of deposition fluxes analysis. Motivated by current scientific relevance, this work explores the chemical characteristics of PAHs in the atmosphere over the coastal urban region of Poland. The main goal of this short-term study was to investigate the chemical composition and deposition fluxes of parent PAHs both in the gas and particulate phase. This study primarily focuses on the variability of 16 PAH isomers, sources and key factors controlling their distribution patterns during the warm study period, which is particularly interesting due to the lack of comprehensive data.

## 2. Materials and Methods

### 2.1. Site Characteristic

The measurement campaigns were performed in Gdynia (54°52′ N, 18°56′ E), which represents a coastal urban region in northern Poland. The sampling site description is presented in Table 1.

### 2.2. Sample Collection and Isolation of PAHs

Air samples for PAH analysis were collected from 20 May–19 August 2021. In this study, the sampling took place for a period of 8 days in each month, which gives a total of 32 daily samples (separately for the particle and gas phases). Particulate samples were collected onto pre-combusted (550 °C, 8 h) quartz fiber filters (QM-A Whatman, Maidstone, UK, 47 mm in diameter). PAH isomers in the gas phase fraction were trapped onto purified polyurethane foam PUF plugs (6 cm OD × 5.1 cm length, Restek, Bellefonte, PA, USA). The low-volume manual sampler Comde Derenda with integrated PM-PUF inlet operated at a flow rate of 2.30 m^3^ h^−1^ was used to collect air samples. In this study, sampling was carried out on the roof of the building (Table 1). The sampling platform was additionally equipped with basic meteorological devices (i.e., air temperature, relative humidity, air pressure, wind speed and direction) to register environmental conditions during the sampling period.

After each daily sampling, samples were transported to the laboratory. In detail, filters were conditioned for 24 h in a desiccator, weighted and then wrapped in aluminum foils, sealed in polyethylene zip-bags and stored frozen until analysis. PUF plugs were placed into zip-lock bags and stored at −20 °C to prevent the loss of volatile/semi-volatile components. The PUF and filter field blank samples were collected each month.

The sample extraction was performed according to an analytical protocol described in the previous laboratory study [23]. Briefly, each sample was solvent extracted in 3 cycles for 15 min in a mixture of dichloromethane (DCM) and *n*-hexane (1:1 in volume, Merck, Darmstadt, Germany) using the ASE350 extraction system (Dionex, Thermo Fisher Scientific, Waltham, MA, USA). High-purity nitrogen was used as a carrier gas. After this step, extracts were purified on a separation column filled with glass wool, 1 cm of anhydrous sodium sulfate (Na_2_SO_4_), 2 g of 2% activated silica gel, and 1 cm of anhydrous Na_2_SO_4_ on top. The column was cleaned by passing through 20 mL of dichloromethane (DCM) and *n*-hexane (1:1 in volume, Merck, Darmstadt, Germany). After this step, 2 mL of samples were injected into the column and eluted with 20 mL of a solution of DCM and *n*-hexane mixture (1:1 in volume). Finally, eluates were concentrated on the rotary evaporator near to 2 mL, then evaporated to 0.5 mL under a gentle stream of nitrogen in a 35 °C water bath, transferred into chromatographic vials, and stored in a refrigerator at 4 °C until further analysis.

### 2.3. HPLC-FLD/DAD Analysis

Samples were analyzed for volatile, semivolatile, and non-volatile PAH compounds using liquid chromatography (Shimadzu Prominence HPLC, Kyoto, Japan) with fluorescence for 15 PAHs: naphthalene (Naph), acenaphthene (Ace), fluorine (Flu), phenanthrene (Phe), anthracene (Ant), fluoranthene (Flt), pyrene (Pyr), benzo(a)anthracene (BaA), chrysene (Chry), benzo(b)fluoranthene (BbF), benzo(k)fluoranthene (BkF), benzo(a)pyrene (BaP), dibenzo(a,h)anthracene (DahA), benzo(g,h,i)perylene (BghiP) and indeno(1,2,3-cd)pyrene (IcdP), and diode-array detections (acenaphthylene (Acy) recorded in 254 nm). The LC column used for PAH separation was a Kinetex (150 mm × 4.6 mm i.d., particle size of 3.5 μm, Phenomenex, Torrance, CA, USA). The gradient elution flow rate was 0.8 mL min^−1^, with acetonitrile and deionized water (1:1, vol/vol) as a mobile phase. The method detection limit (MDL) was calculated as three times the standard deviation of laboratory blanks. The MDLs ranged from between 0.001 ng m^−3^ (BaA) and 0.016 ng m^−3^ (DahA). More details about quality assurance and control of PAH measurements can be found in a previous paper by Siudek [23].

Certified reference materials ERM-CZ100 Fine dust and Chiron (no. 1313.10-100-AN and no. 1328.14-10-AN) were added to samples before extraction to quantify recovery yields and control the method’s reliability. Recoveries of BaA, BaP, BbF, BkF, DahA and IcdP from ERM-CZ100 were 82–110%, while recoveries of 1-fluoronaphthalene and 2-fluorophenantrene surrogate standards from Chiron ranged between 71% and 89%. Replicate analysis (n = 8) of spiked samples (QM-A and PUF) was performed. The PAH concentrations determined in PUF filters were blank corrected.

### 2.4. Meteorological Data

**Figure 1:**
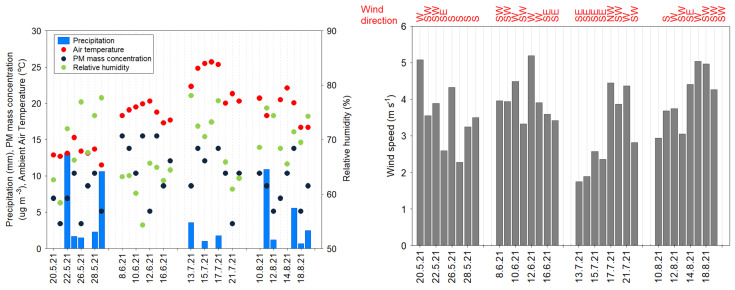
The intra-seasonal variation of basic parameters (precipitation, PM mass concentration, air temperature, relative humidity, wind speed/direction) at the coastal urban site in Gdynia during the 2021 summertime study period.

To investigate the role of meteorological conditions in PAH concentrations’ variability in the gas and particle phase during the summertime study period, the following parameters were considered: T (ambient air temperature), Rh (relative humidity), VD (wind direction), VS (wind speed) and P (daily precipitation). The temporal changes in meteorological factors during the four sampling periods in 2021 between 20 May and 29 May, 8 June and 17 June, 13 July and 22 July and 10 August and 19 August are shown in Figure 1. Note that during the 4-month study period, PM_10_ mass concentration was relatively low (mean of 10.73 ± 8.31 µg m^−3^, with a range of 3.45–51.72 µg m^−3^), due to the fact that the summertime is the less polluted season in this region.

### 2.5. PAHs Deposition Fluxes

The deposition flux of PAHs (*F*, ng m^−2^ day^−1^) for each sampling day was calculated from the following equation:

*F* = *C_PAHi_* × *V_d_* (1)

where:

*C_PAHi_* is the i-analyte concentration (ng m^−3^) in each daily sample

*V_d_* is the deposition velocity of coarse atmospheric particles (m s^−1^). *V_d_* was estimated (1.0 cm s^−1^) using model data from Zhang et al. [24].

## 3. Results and Discussion

### 3.1. Abundance and Composition of Gas and Particle PAH Compounds

Table 2 presents a summary of individual PAH concentrations in the gas and particle phase. During the study period (from May to August 2021), the total mean PAH concentration in the gas phase (26.26 ± 15.83 ng m^−3^) was significantly higher than levels measured in the particle phase (1.77 ± 1.26 ng m^−3^). The highest concentration of 33.92 ng m^−3^ was found for Phe in the gas phase, followed by Flt (15.42 ng m^−3^), Ace (14.22 ng m^−3^) and Naph (5.50 ng m^−3^). The mean concentration of Acy, Ant, BaA and Chry in the gas phase was below 1.0 ng m^−3^.

As shown in Table 2, the peak concentration was observed for Phe (2.39 ng m^−3^) in the particle phase. The Ant and IcdP concentrations in the particulate fraction were in a similar range (0.01–0.43 and <*d.l.*–0.42, respectively). In this research, BaP concentrations ranged between <*d.l.* and 0.30 ng m^−3^, while concentration ranges found for other 5-ring PAHs in the particle phase were as follows: BbF (0.01–0.31), BkF (<*d.l.*–0.17) and DahA (<*d.l.*–0.37). As Table S1 shows, particulate-phase PAHs exhibited positive correlations with PM mass concentrations in June (r = 0.319, *p* < 0.05) and August (r = 0.629, *p* < 0.05), but correlations between these variables were negative and weaker in May and July. This suggests that PM-bound PAHs were affected by local and regional emissions and fluctuations in meteorological factors. Each of these issues will be discussed in the following sections.

**Figure 2:**
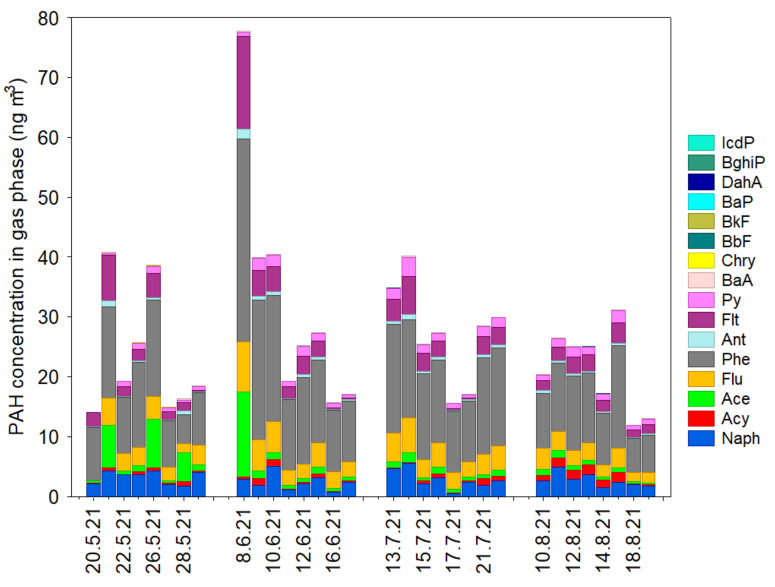
Concentrations of measured PAH isomers in the gas phase during the 2021 summertime study period in Gdynia. All values are significant at the 95% confidence level.

Figure 2 presents the composition profile of PAH isomers in the gas phase. Phenanthrene was reported to be the most abundant aromatic hydrocarbon in the gas phase. The highest concentration of Phe was observed on 8 June. Other PAH congeners were arranged in the following order based on their maximum abundance in the gas phase: Flt > Ace > Flu > Naph > Pyr > Acy > Ant. As exhibited in Figure 2, the peak concentration of 3-ring PAHs in the gas phase showed a slightly different trend to Naph and Acy, implying that this area could be influenced by different primary sources and atmospheric processes during the summertime.

In Gdynia, the Flt/(Flt + Pyr) ratio during the 2021 summertime study period increased in the following order: 0.61 (July) > 0.60 (August) > 0.53 (June) > 0.37 (May), suggesting that particulate-phase PAHs originated from different local/regional sources such as wood combustion and liquid fossil fuels combustion (i.e., traffic emissions). Results showed that traffic emissions were an important source of PAHs in this region. The IcdP to the sum of IcdP and BghiP ratio was the highest in May (0.59), followed by June (0.37), August (0.34) and July (0.33), indicating the influence of gasoline emission and wood-burning [25]. In addition, the BaP/(BaP + Chry) ratio of 0.50 (range: 0.38 to 0.64) suggests primary fossil sources, including liquid fossil fuels combustion, i.e., diesel/gasoline [26].

The mean ΣPAH levels measured in the gas phase in this location were comparable to previous studies, including those determined in coastal stations (i.e., 22.21 ng m^−3^ Aegean Sea; 30.76 ng m^−3^ Marmara—Black Sea) [27], and were higher than concentrations observed at the remote mountain regions in Europe (i.e., 1.3–2.6 ng m^−3^ in the Pyrenees, 2.7–3.7 ng m^−3^ in the Alps and Caledonian mountains) [28] and urban Minnesota, United States (4.1–12 ng m^−3^) [29], but lower than those measured in other urban sites, i.e., Barcelona, Spain (10–36 ng m^−3^) [30], Strasbourg, France (51 ng m^−3^) [9] and Istanbul, Turkey (21–290 ng m^−3^) [31].

### 3.2. Distribution of Particle PAH Compounds

**Figure 3:**
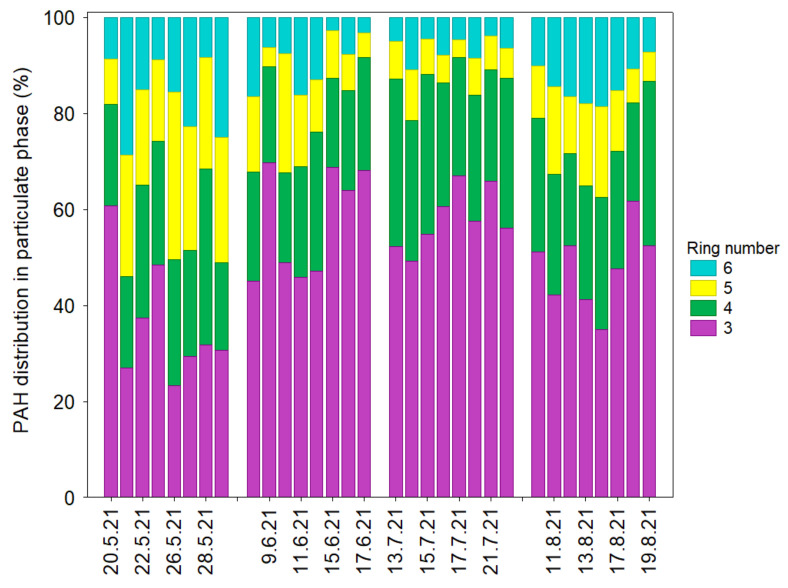
Comparison of the particle-phase PAH components distribution during the study period in Gdynia.

Figure 3 shows the particle phase distribution of PAH congeners during the 2021 summertime study period. The major contributors for the particle phase PAHs were as follows 3-, 4-, 5- and 6-ring compounds, accounting for 50%, 25%, 14% and 12%, respectively. The PAH distribution in the particulate phase was largely affected by weather conditions during the warm study period (Figure 1 and Figure 3). The highest contribution of 3-ring PAHs (Flu, Phe, Ant) accounted for 70% was found on 9 June (P = 0.00 mm, T = 19.1 °C, Rh = 63.4%, VS = 3.9 m s^−1^, VD = SW), followed by 15 June (P = 0.00 mm, T = 18.1 °C, Rh = 64.9%, VS = 3.9 m s^−1^, VD = SW) and 17 June (P = 0.00 mm, T = 17.7 °C, Rh = 64.4%, VS = 3.4 m s^−1^, VD = SE). These findings also showed that the meteorological influences on the particulate matter were similar and PAH emissions were related to local traffic sources, biomass burning, industrial (e.g., petrochemical, refinery) and energy sectors (e.g., power plant). Note that emission from local coal combustion (i.e., boilers, domestic heating units) is significantly lower during summertime compared to wintertime measurements in this region. The 5- and 6-ring PAH components also showed quite different profiles, caused by various pollution sources and individual meteorological factors (Figure 1).

Previous studies [23] showed that traffic emission is one of the largest sources of organic aerosol in this region, with a significant role of 6-ring aromatic hydrocarbons (i.e., gasoline and diesel vehicles). The mean contribution of 5-ring (BkF, BbF, BaP, DahA) and 6-ring isomers (BghiP and IcdP) to the total PAHs increased in the following order: May > August > June > July (Figure 3). The highest 5-ring contribution (26%) to the total PAHs was found on 27 May, while the 6-ring PAHs’ contribution peaked on 21 May. For 4-ring PAHs, the mean contribution in May, June, July and August was 25%, 22%, 29% and 25%, respectively, indicating intra-seasonal differences in emission and atmospheric transformation processes (e.g., gas/particle partitioning) over the coastal urban region of the southern Baltic Sea. The contribution of air pollutants from marine vessels also played an important role in coastal PM chemical composition and PAH levels, especially during days with prevailing wind in NW-SE directions. For example, the relatively high contribution of both 3- and 4-ring hydrocarbons in the particulate phase (>70%) was observed on 25 May (VD = SE, WS = 2.6 m s^−1^), 16 June (VD = SE, WS = 3.6 m s^−1^), 17 June (VD = SE, WS = 3.4 m s^−1^), 13 July (VD = SE, WS = 1.8 m s^−1^), 14 July (VD = SE, WS = 1.9 m s^−1^), 15 July (VD = SE, WS = 2.6 m s^−1^), and 16 July (VD = SE, WS = 2.4 m s^−1^), indicating that in particular low- and medium-molecular-weight organic compounds could be partly attributed to emission from local shipping activities. Recent studies on coastal PM chemical composition in some regions of Asia [32] and Europe [33] also demonstrated that particles of various size modes can be considerably influenced by anthropogenic emissions from sources such as shipping activities.

### 3.3. Gas/Particle Partitioning

**Figure 4:**
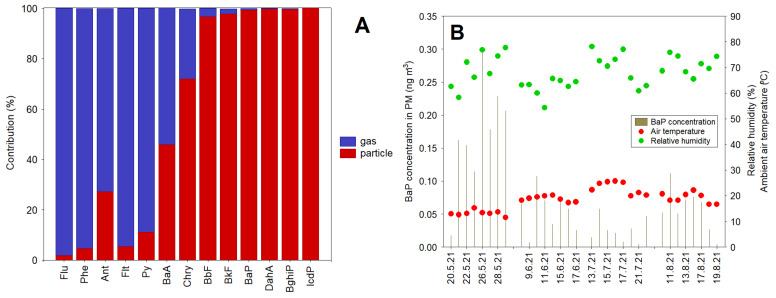
The relative contribution of PAH congeners in the gas and particle phase during the 2021 summertime study period in Gdynia (**A**). Temporal variations in BaP concentrations in PM (ng m^−3^) and meteorological factors (**B**).

To better understand the temperature-dependent atmospheric processes of PAHs, the distribution of individual isomers between the gas and particulate fraction was investigated (Figure 4A). In this study, PAH congeners from Flu to Pyr were found to be predominant in the gas phase (73–98%), while PAHs such as Chry, BbF, BkF, BaP, DahA, BghiP and IcdP were generally higher in the particle phase (72–100%). The distribution pattern of BaA in both phases was relatively similar (46% in the gas phase and 54% in the particulate fraction, Figure 4A). Results from gas/particle partitioning of PAHs in this location were comparable to previous studies [34]. Other studies showed that hot/humid conditions can substantially increase the diffusivity/reactivity and degradation rate of BaP [35,36]. Similar relationships are likely for the summertime study period in Gdynia. It seems that BaP in the particulate phase (Table 1 and Figure 4B) was also included in temperature/Rh-induced atmospheric processes over the coastal region during summer; however, seasonal changes in the BaP profile were mainly caused by local emission effects. When including meteorological data (i.e., ambient air temperature and relative humidity), it was found that BaP concentrations decreased faster during the sampling days with relatively higher air temperatures (i.e., June, July and August) compared to measurements in May, indicating the seasonal significance of changes in BaP degradation rates.

### 3.4. Summertime PAH Deposition Fluxes

**Figure 5:**
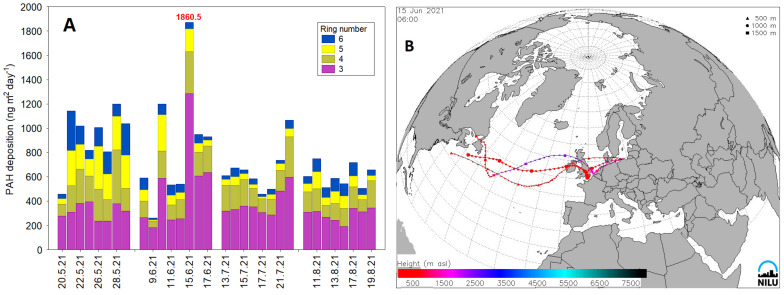
PAH deposition fluxes of 3-, 4-, 5- and 6-ring congeners during the 2021 summertime study period (**A**). The example of backward trajectory on 15 June at 6:00 is calculated using the FLEXTRA model with meteorological data from ECMWF (European Centre for Medium-Range Weather Forecast) reanalysis (**B**).

The variation of PAH deposition fluxes at the coastal urban site in Poland during the 2021 summertime sampling campaign is shown in Figure 5A. The mean ΣPAH deposition flux was 59 ± 24 ng m^−2^ day^−1^ (Figure 5A). The much efficient removal of PM-bound PAHs was typically observed after precipitation events. For example, during the precipitation episode on 25 May, the deposition flux of ΣPAH was 1020 ng m^−2^ day^−1^, while on 26 May, flux accounted for 818 ng m^−2^ day^−1^. For this case, 4-ring PAHs were reduced by 25%, while the reduction of 5- and 6-ring components was much higher and accounted for 32% and 53%, respectively. This suggests that wet scavenging of high molecular weight PAHs was more effective than 4-ring PAHs in this region during warm months. Similar dependency (decrease in deposition fluxes due to wet scavenging of PAHs) was registered on 28 May, 11 August and 17 August, but a slightly different trend was observed on 13 July and 17 July.

Figure 5B shows an example of a typical summertime deposition event on 15 June. The backward trajectory calculation for this case (starting from 00:00 on 14 June to 00:00 on 16 June) clearly showed that the study domain was under the influence of westerly and north-westerly air masses (continental and marine). At the observation time, a peak in daily PAH deposition fluxes (1860.5 ng m^−2^ day^−1^) was observed, coinciding with meteorological conditions, i.e., no precipitation, T = 18.8 °C, Rh = 64.9%, VD = W, VS = 3.9 m s^−1^, PM_10_ mass concentration = 15.52 µg m^−3^. Therefore, it can be concluded that the high deposition of PAHs might suggest mixed local and regional contributions to PAH loadings in this region. Few local sources such as traffic emissions, local coal-fired power plants and docks/ports that are located W–NW from the sampling site could probably have a predominant contribution to PM-bound and gas phase PAHs.

## 4. Conclusions

In the present study, daily variations in concentrations and deposition fluxes of PAHs during warm months were investigated in Gdynia (Poland), which represents the coastal urban area. The main objective was to examine the influence of meteorological factors and sources on the chemical composition of PAHs in the gas and particle phase.

It was observed that summertime differences in 3-, 4-, 5- and 6-ring parent PAH distribution may be influenced by wet scavenging. This finding was supported by statistical analysis, wherein 4-ring PAHs exhibited less effective removal (25%) by daily precipitation than 5- and 6-ring components, whose fluxes decreased by 32% and 53%, respectively.

Based on gas/particle partitioning results, it was concluded that PAH congeners from Flu to Pyr were predominant in the gas phase (73–98%), while PAHs such as Chry, BbF, BkF, BaP, DahA, BghiP and IcdP mostly contributed to the particle phase (72–100%).

These observations (chemical and meteorological) indicate that in the coastal region of the southern Baltic Sea local sources such as traffic emissions and shipping activities probably had a predominant contribution to PM-bound PAHs during the warm study period.

## Figures and Tables

**Table 1:** Characteristic of the sampling site in Gdynia, Poland.

Site	Type	Elevation	Description	Wind Sector
Gdynia (Poland)	Coastal urban	20 m height a.g.l.	Surrounded by maritime ports and docks, ship repair yards.	W-NW, SE
			Shipping emissions: (Baltic Sea), Bay of Puck.	(N), NE-E-SE
			Near buildings, i.e., commercial/residential, domestic heating units, coal-fired heating boilers.	S-SW-W-NW
			Relatively high traffic density.	S-SW-W-NW
			Major industrial sources such as petrochemical refineries, steel manufacturing, coal-fired power plants and municipal solid waste recycling units are about 10–20 km from the sampling site.	SE-S-SW-W-NW

**Table 2:** Atmospheric polycyclic aromatic hydrocarbon concentrations (ng m^−3^) at the coastal urban station in Gdynia (N Poland) during the 2021 summertime study period. The symbol *d.l.* represents concentrations lower than the detection limit.

Compound	Gas Phase			Particle Phase		
	Mean ± 1 S.D. (n = 32)	Min	Max	Mean ± 1 S.D. (n = 32)	Min	Max
**Naph**	2.78 ± 1.27	0.52	5.50	-	-	-
**Acy**	0.62 ± 0.52	0.04	1.70	-	-	-
**Ace**	1.76 ± 2.91	0.25	14.22	-	-	-
**Flu**	3.33 ± 1.54	<*d.l.*	8.45	0.06 ± 0.06	0.02	0.39
**Phe**	13.18 ± 5.74	4.83	33.92	0.65 ± 0.37	0.30	2.39
**Ant**	0.42 ± 0.30	0.09	1.64	0.16 ± 0.12	0.01	0.43
**Flt**	2.89 ± 2.83	0.18	15.42	0.16 ± 0.07	0.05	0.37
**Pyr**	1.17 ± 0.64	0.02	3.24	0.15 ± 0.07	0.04	0.33
**BaA**	0.09 ± 0.07	<*d.l.*	0.27	0.08 ± 0.10	0.01	0.33
**Chry**	0.02 ± 0.01	<*d.l.*	0.05	0.06 ± 0.03	0.01	0.15
**BbF**	-	-	-	0.09 ± 0.08	0.01	0.31
**BkF**	-	-	-	0.05 ± 0.04	<*d.l.*	0.17
**BaP**	-	-	-	0.08 ± 0.07	<*d.l.*	0.30
**DahA**	-	-	-	0.05 ± 0.08	<*d.l.*	0.37
**BghiP**	-	-	-	0.11 ± 0.08	0.03	0.36
**IcdP**	-	-	-	0.09 ± 0.09	<*d.l.*	0.42
**Total**	**26.26 ± 15.83**	**5.93**	**84.42**	**1.77 ± 1.26**	**0.49**	**6.33**

## Data Availability

All data generated or analyzed during this study are included in this paper.

## Supplementary Materials

The following supporting information can be downloaded at: https://www.mdpi.com/article/10.3390/ijerph20054475/s1, Table S1: Correlation coefficient (r) of particulate-phase PAHs (n = 13) and PM_10_ mass concentrations at the sampling site in Gdynia during the 2021 summertime study period.
