# Supplementary material for: Summertime Characteristics of Atmospheric Polycyclic Aromatic Hydrocarbons in a Coastal City of Northern Poland
Source: Int J Environ Res Public Health. 2023 Mar 2;20(5):4475. doi: 10.3390/ijerph20054475 (PMC10001597; doi:10.3390/ijerph20054475)
Supplement: Supplementary file 1 [file ijerph-20-04475-s001.zip › ijerph-2218362-supplementary.pdf]

Supplement of

Summertime characteristics of atmospheric polycyclic aromatic hydrocarbons (PAHs) in a coastal city of northern Poland

*** Patrycja Siudek**

Correspondence to: *Patrycja Siudek (patrycja.siudek@imgw.pl)

Table. S1. Correlation coefficient (r) of particulate-phase Σ_{13}PAHs and PM_{10} mass concentrations at the sampling site in Gdynia during the 2021 summertime study period. All values are significant at the 95% confidence level.

**Variable**	**PAHs_5.21**	**PAHs_6.21**	**PAHs_7.21**	**PAHs_8.21**	**PAHs_summer.21**
PM_5.21	-0.186				
PM_6.21		0.126			
PM_7.21			-0.214		
PM_8.21				0.629	
PM_summer.21					-0.186
